# Use of cyanoacrylate-based surgical adhesives associated to the macroporous tape in skin synthesis in rats^1^

**DOI:** 10.1590/s0102-865020190070000001

**Published:** 2019-09-12

**Authors:** João Ilgenfritz, Ricardo Dutra Aydos, Iandara Schettert Silva, Luiz Carlos Takita, Antônio Carlos de Abreu, Cynthia Alexia Cunha Silva, Karina Bossi Faleiros, Evair Moisés de Lima Santiago, Rondon Tosta Ramalho

**Affiliations:** I MD, Universidade Federal do Mato Grosso do Sul (UFMS), Campo Grande-MS, Brazil. Conception and design of the study, technical procedures, analysis and interpretation of data, manuscript writing.; II PhD, Associate Professor of Surgery, Postgraduate Program in Health and Development in the Midwest Region, UFMS, Campo Grande-MS, Brazil. Conception and design of the study, interpretation of data, final approval.; III Full Professor, Postgraduate Program in Health and Development in the Midwest Region, UFMS, Campo Grande-MS, Brazil. Intellectual and scientific content of the study.; IV PhD, Assistant Professor, Department of Surgery, UFMS, Campo Grande-MS, Brazil. Histopathological examinations.; V Fellow Master degree, Postgraduate Program in Health and Development in the Midwest Region, UFMS, Campo Grande-MS, Brazil. Technical procedures.; VI Fellow Biologist undergraduate, Biosciences Institute, UFMS, Campo Grande-MS, Brazil. Technical procedures.; VII Fellow medical undergraduate, Faculty of Medicine, UFMS, Campo Grande-MS, Brazil. Technical procedures.; VIII Full Professor, Postgraduate Program in Health and Development in the Midwest Region, UFMS, Campo Grande-MS, Brazil. Technical procedures, macroscopic and histopathologic analysis, manuscript writing.

**Keywords:** Adhesives, Cyanoacrylates, Rats, Wistar

## Abstract

**Purpose:**

To compare the use of new cyanoacrylate surgical adhesive associated with macroporous tapes in cutaneous synthesis.

**Methods:**

Male Wistar rats with a longitudinal incision of 4cm were used on the back, divided into four groups: GI used octyl-cyanoacrylate (Dermabond^®^), GII used N-2-butylcyanoacrylate, GIII used octyl-cyanoacrylate and macroporous tape and GIV used N-2-butyl cyanoacrylate and macroporous tape. On the fourteenth day, the rats were submitted to euthanasia, were divided in two parts, and a layer of skin subcutaneous tissue through an area of operative healing was removed. One part was submitted to the study of rupture strength with the use of tensiometer, and in the other part histological examination was performed.

**Results:**

No force test was similar between groups I and II, being different from groups III and IV (P <0.001), which were identical to each other (P> 0.05). The units were compared among the studied groups, and they were different with the use of macroporous tapes (P> 0.05).

**Conclusions:**

The purpose of macroporous tapes is associated with CA adhesives in cutaneous tissues that provide more resistant scars. The use of a combination of macroporous tapes leads to complete re-epithelialization, without provoking foreign body reaction, has hemostatic properties and does not cause an absorptive reaction.

## Introduction

The wound healing process is a sophisticated mechanism dependent on several factors, including the extent and depth of an injury, which will determine a cicatricial response, and the more complicated or time-consuming wound occlusion will increase the chances of developing a wound of worse quality^1^.

Tissue damage of any nature (physical, chemical or biological) immediately triggers a series of events resulting from the activation of cells in the body by chemical and physical stimulation. Fragments of the inert elements of the tissue such as collagen, elastin, fibronectin extravasate from ruptured vessels and by the action of inflammatory mediators culminate with the beginning of the tissue repair process^2^.

Research involving wound closure techniques has evolved a lot in recent years, leading to the emergence of several works involving synthetic and absorbable sutures, the use of staplers, tapes, and other methods. The appearance of surgical adhesives fit into the new forms of wound closure and has been studied for approximately four decades^3^.

The healing process is dynamic and involves biochemical and physiological events to guarantee tissue repair. It is didactically divided into three phases, taking into account its macroscopic and histological aspects in inflammatory, fibroblast, and remodeling^2^.

The process of angiogenesis or growth of new vessels is intimately involved with the physiological process of healing; neovascularization acts as a source of vital nutrients and a drainage pathway for products to be discarded, contributing to the formation of granulation tissue^4^.

Surgical adhesives have some advantages such as technical ease, shortening the surgical time, as it is not necessary the introduction of the foreign body; they also decrease the time of recovery of the wound, resulting in a minor inflammatory reaction and a better tissue synthesis, besides being an alternative to conventional sutures^5^.

Usually, these compounds have been used as complements to conventional sutures, being their use isolated and, when used, presenting questionable results^6^.

They have unique properties including bacteriostatic, hemostatic, biodegradable, and biocompatible characteristics, except for methyl cyanoacrylate. They are easily manipulated but have some drawbacks, such as: less resistance in areas of tension when compared to conventional sutures, and toxicity in some patients. One of the most important advantages is the ease of manipulation compared to other synthetic methods such as sutures^7^.

The discovery of industrial adhesives based on cyanoacrylate occurred in 1949 by ARDIS, and its effect began to be studied in tissues; however, its application in humans was only carried out in 1960. They were found to be useful in plastic surgery, neurosurgery, cardiac and dental surgeries. These adhesives have a remarkable polymerizability when exposed to water or blood. This reaction results in immediate occlusion of small vessels. Since then, cyanoacrylate compounds have been used in the embolization of aneurysms, vascular malformations, and lacerations. These adhesives degrade in formaldehyde and cyanide that are known to be toxic to the body, but so far there is no evidence of toxicity before releasing with topical use^8^ ^,^ ^9^.

The first adhesives to be produced for use in medical practice were composed of shorter side chains (methyl and ethyl cyanoacrylate) which made them more susceptible to breakage, so their use was restricted to low tensile situations. Increased tensile strength was achieved with the use of higher alkyl chains and showed decreased toxicity by retarding degradation. For this reason, the most used in medicine are N-butyl-2-cyanoacrylate (Indermil^®^), 2-octylcyanoacrylate (Dermabond^®^) and butyl-2-cyanoacrylate (Histoacryl^®^), which have larger chains^10^.

Since the introduction of Dermabond^®^ in the market in Germany, several studies have shown that if used correctly, the cosmetic result is equal to or higher than sutures, with similar rates of infection and dehiscence. Dermabond^®^ is a cyanoacrylate adhesive that forms a layer on the wound keeping the edges close, allowing the healing process. It can be used to replace sutures with 5-0 or smaller wires in incisional wounds or lacerations and is also water resistant^11^.

When in contact with moist tissues, the CA initiates a rapid polymerization reaction promoting instant adhesiveness with a strong bond to the applied tissue promoting efficient hemostasis^12^.

Since, because of the high cost that these AC patches have, their use becomes impaired in medical practice even though studies have shown their benefits. The purpose of this study was to evaluate a new surgical adhesive composed of N-2-Butylcyanoacrylate produced by the chemistry laboratory of the Federal University of Mato Grosso do Sul Foundation (UFMS) in a cooperation agreement with a private company under protocol number 23104.001269/2010-21 -UFMS where this adhesive could be produced and used locally.

## Methods

This experimental research was evaluated by the Animal Use Ethics Committee (CEUA) of the Universidade Federal do Mato Grosso do Sul and all procedures were performed according to the Animal Experimentation Ethics Committee under protocol number 882/2017.

### Experimental design

Thirty-two male Wistar rats (*RattusNorvegicus Albinus*), weighing between 250 and 350 g, were used from the central laboratory of UFMS.

The rats were housed in polypropylene boxes, suitable for the species (50×40 cm), with one animal per box; receiving appropriate ration for the species (Nuvilab^®^, NuvitalNutrientes Prod. Vet. Ltda - Curitiba - PR) and water at will. They remained in a controlled environment, in light / dark cycles of 12 hours, with a stable temperature (23 + 1°C) maintained by air conditioning without restrictions in the movement. The animal wastes and boxes were replaced every 48 hours.

The animals were divided into four groups with eight animals each.

GI - A 4-cm cut incision and cutaneous synthesis was performed using octyl-cyanoacrylate (Dermabond^®^).

GII - An extensive 4-cm cut incision and synthesis with a surgical adhesive composed of N-2-Butyl cyanoacrylate was performed.

GIII - It was performed a wide trichotomy and a skin incision of 4cm in the back and cutaneous synthesis using macroporous tape to approach the edges of the wound and synthesis with octyl cyanoacrylate (Dermabond^^®^^).

GIV - It was performed a wide trichotomy and a cutaneous incision of 4 cm in the back and cutaneous synthesis using macroporous tape to approach the edges of the wound and synthesis with N-2-Butyl cyanoacrylate.

The amount of adhesive used for all groups was standardized in two drops for each centimeter of incision, totaling eight drops per animal.

After the procedure, the animals were housed again in polypropylene boxes respecting the separation of the groups, leaving the GI with 8 animals and analysis in 14 days, GII - with 8 animals and analysis in 14 days, GIII with 8 animals and analysis in 14 days; and GIV with 8 animals and analysis in 14 days.

### Procedures

For anesthetic induction, the animals were weighed and anesthetized with intradermal injection of a 2:1 solution of Ketamine Hydrochloride (Cetamin^®^), 50mg/ml, and Xylazine Hydrochloride (Xilazin^®^), 20mg/ml, respectively, in the dose of 0.1ml/100g.

The trichotomy, scraping and cutting off the animals back furs followed was by mechanical cleaning with a soft bristle brush embedded in 1% polyvinyl pyrrolidone iodine and rinsed with 0.9% sodium chloride solution followed by antisepsis of the back with alcohol solution iodinated 2%.

A 4-cm incision was made in the skin and subcutaneously on the back in all groups, using the last rib as the caudal margin and the spine as the dorsal margin.

After the 14-day observation period (Groups GI-14, GII-14, GIII-14, and GIV-14) the animals underwent euthanasia by deepening the anesthetic plane, then each rat was immobilized on the surgical table and a skin segment and subcutaneous cellular tissue of approximately 5×3cm were removed and divided into cranial and caudal ends. One part was taken directly to the freezer at a temperature of −20°C to be submitted later to study of the rupture strength; and another was fixed immediately in 10% buffered formaldehyde for 48 hours and was HE staining prepared for identification of inflammatory reaction by the presence of mono- and polymorphonuclear cells, foreign body type giant cells, epithelization, and hemorrhage^13^.

### Tensile strength test

Samples for the rupture strength study were taken frozen for submission to the tensile test in the mechanical test laboratory (SENAI, Teresina - PI), using the test machine DL 20000 (EMIC) with manually adjustable grippers and electronic data acquisition system with Tesc software version 3.04.

The segments of the healing line were attached to aluminum claws parallel to the synthetic line and these claws connected to the apparatus that exerted the tensile force perpendicular to the scar. The values were given in kilogram-force and turned into gram-force. The rupture strength corresponds to the most significant force value required during the sample traction, i.e., the highest strength value of the sample to achieve rupture. The traction occurred at a speed of 50mm/min, and the traction force was continuously measured automatically ^14^.

### Statistical analysis

For statistical analysis, a description of the results found for the tensiometric and histological values was carried out, besides the analysis of the four groups.

For analysis and comparison of rupture strength found in each group, the Tukey's analysis of variance was applied for multiple comparisons.

For the histological analysis of the blades stained with HE, the chi-square test applied to the experimental groups was used. The level of significance was set at α=0.05.

## Results

### Clinical evolution

**Figure 1:**
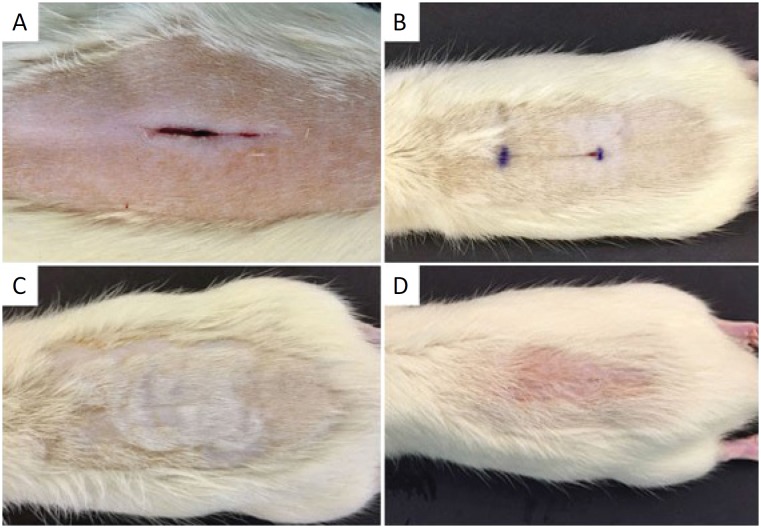
Macroscopic aspect of cicatrization after 14 days. **A,** total dehiscence of synthesis and cicatricial retraction observed with the use of CA. **B,** Octyl cyanoacrylate synthesis. **C**, synthesis with N-2-butyl cyanoacrylate. **D**, synthesis with N-2-Butyl cyanoacrylate + macroporous tape.

There were no deaths before the euthanasia at 14 days. In the control groups (I and II) total dehiscence was observed in group I (12.5%) and two in group II (25%). Regarding experimental groups (III and IV), total dehiscence was not observed in group III and only one in group IV (12.5%) (Fig. 1).

Due to the rapid growth of the furs, the experimental groups that received macroporous tapes showed an early detachment of the adhesive with about six days of the experiment, leading to small regions of partial dehiscences.

### Biomechanical analysis

Table 1 shows the results of the descriptive analysis of the results found in each group.

**Table 1:** Results found for rupture strength values after 14 days of cutaneous synthesis.

	Groups			
	Group I (mean^+^/_SD)	Group II (mean^+^/_SD)	Group III (mean^+^/_SD)	Group IV (mean^+^/_SD)
Tension (g)	601,4 ± 87,5	742,2 ± 135,8	1001,8 ± 143,1	1120,0 ± 173,3

The D’Agostino and Pearson normality test indicated a Gaussian distribution of the data, which is why variance analysis was chosen. Significant differences were found between the experimental groups (p <0.001) and the Tukey's test was applied for multiple comparisons between groups. The level of significance was set at α = 0.05.

**Figure 2:**
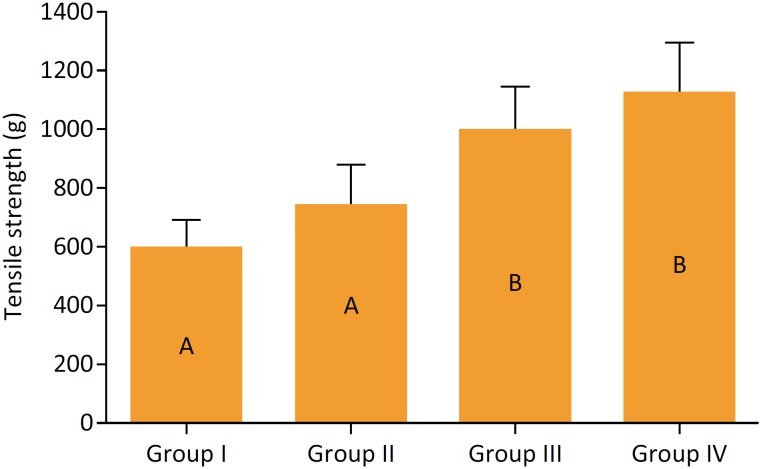
Mean of the rupture values found in the groups. The vertical line represents the standard deviation. Unequal letters denote statistically significant differences (α = 0.05).

The complementary test indicated statistical similarities between groups I and II, which were different from groups III and IV (p <0.001), which were similar to each other (p> 0.05) (Fig. 2).

### Histological analysis

**Figure 3:**
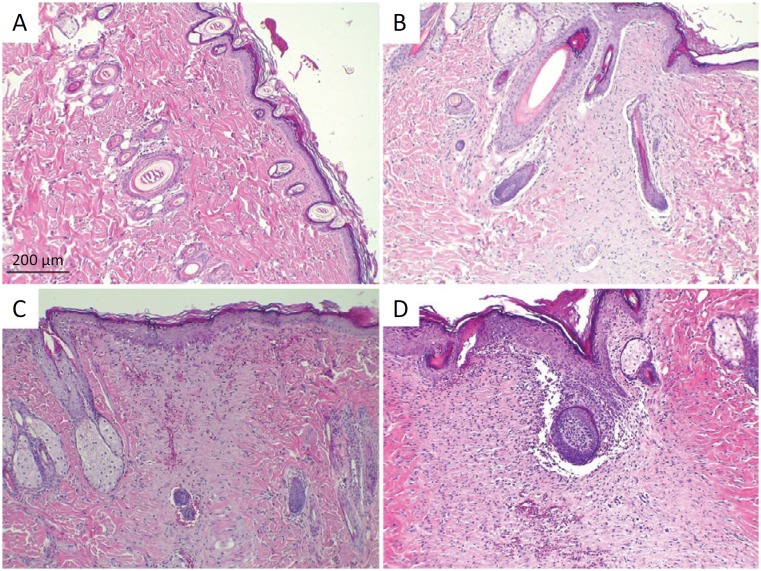
Photomicrography of epithelial tissue stained with HE. **A**, group I. **B**, group II. **C**, group III. **D**, group IV.

Histological sections of Group I skin demonstrate stratified keratinized squamous epithelial tissue (re-epithelization) in all animals. The dermis exhibited proliferation of fibroblasts with the presence of connective tissue in the organization and the presence of some adjacent structures (fur follicle). The Dermis of a skin fragment exhibited a focal area of hemorrhage (Fig. 3a).

In group II, histological sections of the skin demonstrate an absence of keratinized squamous epithelial tissue in two animals (ulcer), with the presence of necrotic debris and inflammatory cells (predominantly represented by activated macrophages and few neutrophils). In five animals there was complete re-epithelialization; however, with a thin layer of epithelial cells of the epidermis. The dermis exhibits proliferation of fibroblasts with the presence of connective tissue, collagen fibrils in organization and presence of few adjacent structures (fur follicle) (Fig. 3b).

In group III, the absence of keratinized squamous epithelial tissue was observed in one animal (ulcer), with the presence of necrotic debris and inflammatory cells (predominantly represented by activated macrophages and few neutrophils). In four animals there was complete re-epithelialization; however, with a thin layer of epithelial cells of the epidermis. The dermis exhibits proliferation of fibroblasts with the presence of connective tissue, collagen fibrils in organization and the presence of few attached structures (fur follicle). It was observed that in three animals regeneration was complete in all layers (Fig. 3c).

The histological sections of the skin in group IV demonstrate the absence of keratinized squamous epithelial tissue in two animals (ulcer), with the presence of necrotic debris and inflammatory cells (predominantly represented by activated macrophages and few neutrophils). In four animals there was complete re-epithelialization; however, with a thin layer of epithelial cells of the epidermis. The dermis exhibits proliferation of fibroblasts with the presence of connective tissue (connective) collagen fibrils in organization and the presence of few attached structures (fur follicle). It was observed that in two animals regeneration was complete in all layers (Fig. 3d).

**Figure 4:**
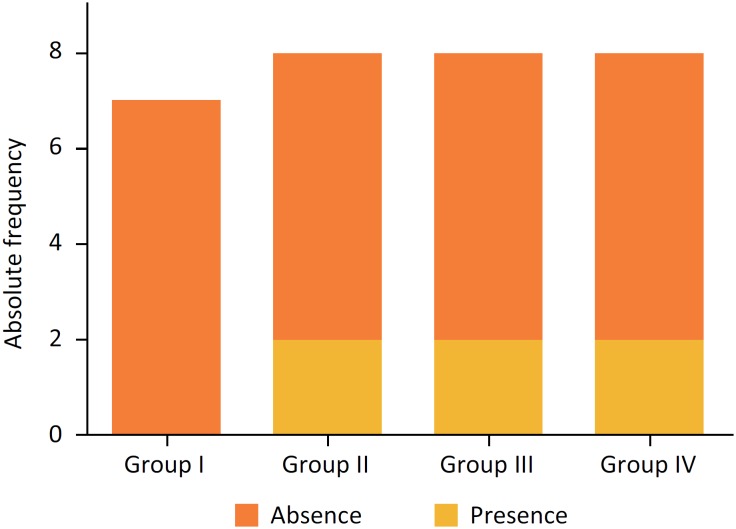
Presence or absence of inflammation after staining of HE in the groups studied.

In the inferential analysis, there were no differences between groups regarding inflammation (p = 0.538), re-epithelialization (p = 0.556), ulcer (p = 0.502) and hemorrhage (p = 0.558) (Fig. 4, Table 2).

**Table 2:** Results found for histological parameters after 14 days of cutaneous synthesis.

Microscopic Findings	Experimental groups			
	Group I	Group II	Group III	Group IV
**Inflammation**	0	2	2	2
**Re-epithelialization**	Total	6/8	7/8	7/8

**Strangebody**	absent	absent	absent	absent

**Ulcer**	absent	2/8	1/8	2/8

**Attached Glands**	P	P	P	P
**Hemorrhage**	1/8	0/8	1/8	0/8

Score: 0 (absent), 1 (slight-25%), 2 (moderate-50%), 3 (intense -75%) e 4 (severe -100%).

## Discussion

This experiment showed a similar index to the short-term dehiscence that can be observed in the literature, demonstrating that the adhesive is less efficient in areas of tension than the conventional suture, limiting its isolated use to areas of low tension. Ilgenfritz *et al*.^14^, when performing a similar experiment with the same N-2-butyl cyanoacrylate and Octyl-cyanoacrylate adhesive, found a total of 33.3% dehiscence with Octyl-cyanoacrylate alone and 41.6% with N-2-butylcyanoacrylate 14, and in this experiment 12.5% dehiscence was observed in group I and 25% in group II, groups where the adhesives were used in isolation.

When analyzing the rupture strength of the animals submitted to cutaneous synthesis, we found that both groups I and II had significantly weaker scars with 14 days of evolution compared to groups III and IV, but similar to each other, whereas groups III and IV, where a greater rupture strength was performed, also were similar to each other. Such fact demonstrates that the use of adhesives in areas of tension should not be done in isolation and that the macroporous tape helped to maintain the coaptation of the wound edges.

In a study, Singer *et al*.^15^ analyzed 20 papers and suggested the concomitant use of porous adhesive tapes to facilitate the coaptation of the edges and reinforce the suture.

The use of the adhesive should be limited to uninfected or contaminated lesions, places of low tension, traumatic or surgical wounds, as long as there is no difficulty in coaptation of the borders. In regions of fragile skin, it presents advantages concerning the threads since it does not cause injury or ischemia^8^.

Despite the many advantages documented by CA adhesives, their use remains restricted due to their limited use in voltage areas. Using *CaviaPorcellus,* Noordzij *et al*.^16^, found a significant difference in operative wound tension immediately after the repair. In the suture group, there was a tension force 12 times greater than the adhesive group, but after seven days, there was no difference in the rupture strength. As the test was performed immediately after the experiment, being tested only the synthesis methods, there was no time for a proper wound healing process with collagen and scar tissue to occur.

Souza^17^, when evaluating 153 patients with facial lesions over a 12-year period, where synthesis was performed using CA adhesive, found 5.6% dehiscence and 13.9% unsightly scars, attributed to local characteristics such as skin mobility, areas of tension and inflammatory reaction linked to the subdermal sutures. In this experiment we found more resistant scars in the experimental groups (III and IV) with 14 days of evolution compared to groups I and II, showing that the use of the macroporous tape associated with the adhesives is an essential addition to the synthesis methods resulting in more scarring resistance and with a lower rate of dehiscence.

Ilgenfritz *et al*.^15^, when evaluating dorsal cutaneous synthesis in 36 Wistar rats, found no difference between the healing strength in animals where the synthesis was directly performed with Dermabond^®^ and N-2-butylcyanoacrylate (same adhesive used in this research) after seven days of evolution. It is worth noting that the methodology was similar to this experiment, with manual approximation and subsequent application of the adhesives. In this work, statistical equivalence was found between the groups where the adhesives were used in isolation (I and II), corroborating with the data already presented in the literature^14^.

Adhesives AC are indicated for low-tension surgical sites and traumatic lacerations where the edges of the wound easily approximated. There are several reports of the successful use of these adhesives in the sealing of nephrostomies and spinal fluid leakage. They should be avoided in mucosal areas and regions with high humidity or attrition, as continuous exposure to water induces premature detachment of the adhesive. They should not be used alone for the closure of wounds with high tension but can be used in this regions when associated with sutures to reduce local tension or in conjunction with adhesive tapes^15^.

Samples taken for analysis of rupture strength were immediately taken to the freezer at −20°C and transported frozen to the laboratory for testing. According to Tognini *et al*.^18^, the freezing rate directly influences the quality of the material and should be as fast as possible, thus reducing micro organic and enzymatic modifications. There is no significant difference in the rupture strength and the deformation of segments when comparing storage methods in rats submitted to laparotomy followed by laparorrhaphy, when compared to the rupture strength in the surgical scar immediately after euthanasia, after preservation for 20 days in a freezer at -17ºC and after maintenance in carbon dioxide for 20 days.

In this experiment, the collected samples were taken directly to the freezer, and later they were submitted to the proposed test, following the parameters given by the already exposed literature.

These adhesives have advantages over conventional sutures for the skin and mucous membranes and are indicated for a variety of syntheses such as gingival lesions, skin and mucosa lacerations, with excellent biocompatibility and few secondary tissue reactions. compatible with injuries on the back of rats as used in this research, with higher tension^6^.

Post-surgical complications of CA, such as inflammation and dehiscence, are similar to those seen with conventional suture in children undergoing low-tension surgical incisions. The adhesive, when applied continuously at the edges of the wound, blocks the path for vessel growth, and when used in excess, can cause thermal damage to the surrounding tissues. It is worth mentioning that the wounds induced in this experiment were submitted, firstly, to a manual approximation of their edges for posterior placement of the surgical adhesive^19^.

Studies reviewed have shown good results with the use of cyanoacrylate adhesives for human skin suture, comparable to traditional suture methods. Prospective studies have been done using CA adhesives, demonstrating better results concerning wound healing and scarring over conventional sutures^20^ ^,^ ^21^.

Factors such as VEGF, FGF, angiopoietin and TGF-β stimulate endothelial migration. In addition to these soluble factors, two structural characteristics were shown to be fundamental for endothelial migration. First, there must be an extracellular matrix present between the edges of the wound. Second, there must be an absence of any foreign substance nearby, known as the effect of free margins. For these cells to navigate over the ECM, they must express integrins and metalloproteinases^22^.

Costa^23^, when comparing the number of neovascularization in the scar of rabbits submitted to abdominoplasty did not find a significant difference between animals submitted to cutaneous synthesis with nylon and octyl-2-cyanoacrylate after 14 days of evolution, finding only a higher amount of eosinophils in all scars where cyanoacrylate adhesive was used.

In an experimental study, Costa *et al*.^24^ comparing the use of butyl-2-cyanoacrylate, suture and gelatin-resorcinol-formaldehyde (GRF) blend in the stabilization of cartilage grafts in rabbits, found a more significant inflammatory process in the three groups in the first two weeks decreasing up to 12 weeks. Furthermore, they found no significant difference between groups of animals of 2, 6 and 12 weeks concerning inflammatory infiltrate, angiogenesis and degree of fibrosis when compared to the different types of fixation. In this experiment, when evaluating the four groups of animals, we observed a moderate inflammatory process represented by activated macrophages and few neutrophils in groups II, III and IV, and not in group I. In spite of this alteration, the adhesives showed to be similar to each other, and the association with macroporous tapes did not modify the inflammatory parameters analyzed.

The compact dermis lies beneath the epidermis and is formed by dense, modeled connective tissue made up of thick collagen fibers, corrugated and parallel to the surface of the epidermis. The loose dermis is located below the basement membrane with thinner collagen fibers and blood vessels^25^. In this study, a proliferation of fibroblasts with the presence of connective tissue and collagen fibrils in organization was observed in the analysis of the dermis after HE staining. This result was similar in all studied groups, showing a similarity between the adhesives analyzed; and even though the association with the macroporous tape produced more resistant scars, it did not demonstrate significant histological differences between the groups^26^.

When comparing N-Butyl-2-cyanoacrylate with polyglycolic acid sutures in the closure of dorsal wounds in rabbits, Pelisser *et al*.^27^ demonstrated the persistence of the cyanoacrylate film on the base and wound margins at histological evaluation after two weeks. It was found a medium and focal acute inflammatory reaction with diffuse edema of multinucleated giant cells similar to the foreign body reaction. In the suture group, a medium inflammatory reaction occurred, with giant cells around the wire. The authors concluded that n-butyl-2-cyanoacrylate did not delay or inhibit the healing process or its quality, besides the speed and ease of application^19^. In this experiment we found similar results concerning the inflammatory reaction with the different adhesives studied, being these identical to each other with or without the use of macroporous tapes. No foreign body reaction was observed in any animal studied, and re- epithelialization was complete in all animals in group I, in six animals in group II and in seven animals in groups III and IV (Table 1). This demonstrates equivalence between groups. The manual approach of the wound edges for subsequent application of the adhesives as performed in groups I and II was equivalent to the use of macroporous tapes (groups III and IV) to maintain coaptation, despite the association of the tapes to lead to more resistant scars.

In an experimental study using 15 *Rattus Norvegicus,* Saska^28^, observed an immediate hemostatic effect with butyl cyanoacrylate, foreign body type reaction with visualization of giant cells in HE staining adjacent to Superbonder^®^ and Histoacryl^®^ adhesives in the period 7 and 30 days when performing dorsal incisions in the animals and synthesis with the mentioned adhesives. He also observed a mild inflammatory reaction with the adhesives in the first seven days and a more intense reaction when compared with the suture group, justified by surgical trauma due to the penetration of the needle and thread. In this study, at 14 days, bleeding focus in only one animal in groups I and III was observed, where Dermabond^®^ was used. Bleeding in groups II and IV where N-2-Butyl cyanoacrylate was used, was not found, showing a statistical equivalence between the two products. These data corroborate the hemostatic effect of cyanoacrylate adhesives, being an essential advantage over suture stitch synthesis.

The use of CA has some disadvantages such as its high cost, less availability, less resistance to tension, and humidity. In this experiment, an N-2-Butyl cyanoacrylate adhesive was used in the UFMS chemistry laboratory with good availability for local use, facilitating the use of these adhesives in the medical practice^9^.

## Conclusions

The use of CA adhesives associated with macroporous tapes produces more resistant scars when compared to their isolated use.

The adhesives studied are similar concerning the resistance of the scars produced.

Both the isolated use of the studied CA adhesives and their association with macroporous tapes lead to complete re-epithelialization, do not provoke foreign body reaction, have hemostatic properties, and do not cause inflammatory reaction.
